# Supplementary material for: SARS-CoV-2 spike and nucleocapsid proteins fail to activate human dendritic cells or γδ T cells
Source: PLoS One. 2022 Jul 14;17(7):e0271463. doi: 10.1371/journal.pone.0271463 (PMC9282473; doi:10.1371/journal.pone.0271463)
Supplement: Attachment — Submitted filename: Singh et al. PLoS One - responses to reviewers.docx [file pone.0271463.s002.docx]

**Manuscript number: PONE-D-22-10090**

**PLoS One**

**Responses to reviewers**

SARS-CoV-2 spike and nucleocapsid proteins fail to activate human dendritic cells or γδ T cells

Kiran Singh, Sita Cogan, Stefan Elekes, Dearbhla M. Murphy, Sinead Cummins, Rory Curran, Zaneta Najda, Margaret R. Dunne, Gráinne Jameson, Siobhan Gargan, Seamus Martin, Aideen Long, Derek G. Doherty

Matthias Eberl, Ph.D.

Academic Editor

PLOS ONE

Dear Dr. Eberl

We are very grateful to the reviewers and editors for providing us with critiques of our manuscript. We have addressed all points below in blue text and revised the manuscript accordingly. All additions to the text are indicated with yellow highlighting in the revised manuscript and the locations of these changes are indicated in our responses below. We feel that our paper has improved as a result of these revisions.

Reviewer #1: This manuscript addresses a clear knowledge gap with a logical sequence of robust experiments from which appropriate conclusions have been drawn. There is considerable interest in the role of γδ T cells in mucosal inflammatory disorders, particularly in the context of COVID-19 and the development of new drugs to combat this. It is therefore important to understand the mechanisms that drive γδ T cells activation and recruitment to the lung. In this report, the authors present strong evidence that γδ T cells are not directly activated by the major viral spike or capsid proteins, which has obvious implications for efforts to target this population in the clinic. The findings are technically sound, described clearly, and represent an important contribution to the growing body of knowledge in this area.

Thank you for this encouraging review of our manuscript.

Reviewer #2: This manuscript investigates whether proteins or peptides from SARS-Cov2 spike and nucleocapsid activate Vd1 or Vd2 T cells. While it is known that gd T cells in humans respond and participate in the immune response to SARS-Cov2, it is still unknown how they become activated. While the data here represent a start to this endeavor, there are very few conclusive findings in the manuscript in it's current form.

1. There is little investigation or discussion into the very possible point that the Vd2 T cells respond to upregulation of IPP upon virus infection of neighboring cells. Direct viral protein activation in human gd T cells is not a common mechanism of activation, so seems a strange hypothesis. Activation of gamma delta T cells by isoprenyl phosphate molecules has been carefully examined with regard to influenza virus and may be a stronger hypothesis here, especially with the negative findings.

We thank you for this suggestion and have now included a section on the possibility that isoprenyl pyrophosphate produced by SARS-CoV-2 infection can activate Vδ2 T cells and have used the influenza studies to illustrate this point. This is highlighted in the revised discussion (page 16, last 3 lines and page 17, first 3 lines)

2. There was contamination of the proteins used in the study negating the results from several figures. These results should not be included.

We agree that contamination of the RB-nucleocapsid protein negates the results from some figures, but we have not removed these from the figures because it illustrates an important methodological issue when using recombinant proteins to stimulate DC; because the results are useful in showing that DC activation leads to downstream activation of γδ T cells (final paragraph of the discussion – pages 18, 3^rd^ paragraph, lines 4-8); and because the other reviewers liked these figures.

3. There was no positive control to support spike or nucleocapsid proteins being present or at the proper concentrations etc.

The same batch of recombinant spike protein was used by our colleagues to set up a SARS-CoV-2 serological assay and its concentration is fully validated in their publication (Phelan et al. 2021, Viruses 13: 1371, doi: 10.3390/v13071371). Both batches of the nucleocapsid were similarly validated by co-authors of the present manuscript Zaneta Najda and Seamus Martin. A range of concentrations of both proteins were used to stimulate conventional T cells within PBMC and 1 μg/mL was found to be optimal (Figure 2). We therefore used this protein concentration to stimulate DC and γδ T cells.

4. The findings are minimal and more studies need to be performed to come to any conclusions.

It is true that the findings are negative, but they contrast with two published studies that found that spike protein can bind to TLR4 on macrophages, and they clearly rule out roles for spike and nucleocapsid proteins in activating DC and γδ T cells.

Reviewer #3: This manuscript concludes that Spike protein and NP from SARS-Cov2 do not result in activation of d1 or de gd T cells, whether directly or through DC. Minor DC-mediated activation is hypothesised to be through contaminant, such as viral RNA. The paper is technically ok, well written and the data well described. Whilst the conclusions from the studies performed are correct there are questions as to the relevance of these in a physiological in vivo context: gdT cells (d1 as well as d2) sense infected cells (viral as well as bacterial) and whilst the authors point this out in the discussion, they did not address this by studying gdT reactivity following exposure to infected cells, DC or other.

We were unable to carry out these experiments using virus-infected cells, due a requirement in our institution to carry out experimentation with live SARS-CoV-2 in a biosafety level 3 facility. We agree that such experiments would have important implications for the relevance of γδ T cells in SARS-CoV-2 infection in vivo. We have now re-written the discussion (pages 15-19) to highlight this issue and to speculate on how γδ T cells may be activated by SARS-CoV-2 infection in vivo.

The other possible means by which gdT cells can be activated is if exposed to antibody opsonised virus or infected cells. Such Ab and target can then bind to FcR (CD16) on the gdT cells and lead to gd T activation and target uptake. This was not studied or even mentioned as possibility. These two points diminish the value of the work.

We thank you for raising this possibility as to how SARS-CoV-2 may activate γδ T cells and have now included this in the introduction (page 4, 2^nd^ paragraph, lines 4-5) and the discussion (page 1, 2^nd^ paragraph, last 5 lines) of the revised manuscript.

No minor points - very good figures, legends and text.

Editor’s comment

In particular, a revised manuscript should place a stronger emphasis on the physiological relevance of the experiments performed and the underlying hypothesis of a direct action of human γδ T cells by viral proteins, considering alternative mechanisms by which human γδ T cells might be activated in the context of a SARS-CoV-2 infection.

We have now re-written the discussion (pages 15-19) to emphasise the physiological relevance and shortcomings of our study and have speculated on the various mechanisms by which Vδ1 and Vδ2 T cells may be activated in SARS-CoV-2 infection.
